# Optimizing COVID-19 vaccination strategies for high-risk populations: potential and challenges of combining heterologous boosting with respiratory mucosal delivery

**DOI:** 10.3389/fpubh.2026.1849650

**Published:** 2026-06-12

**Authors:** Ruixia Miao, Jing Wang, Yuan Huang, Xiaolu Zhang, Pengfei Lei, Dehong Li, Che Chen, Yan Lu

**Affiliations:** 1 School of Public Health, Gansu University of Chinese Medicine, LanZhou, China; 2 Department of Blood Transfusion, Gansu Provincial Hospital, LanZhou, China; 3 Department of Clinical Laboratory, Gansu Provincial Hospital, LanZhou, China

**Keywords:** heterologous boosting, high-risk populations, mucosal immunity, respiratory mucosal delivery, SARS-CoV-2

## Abstract

Despite the decline of the global peak of COVID-19, SARS-CoV-2 continues to circulate, evolve, and undergo immune escape, posing persistent threats, particularly to older adults, individuals with comorbidities, and immunocompromised populations. Although existing vaccination strategies have substantially reduced the risks of severe disease, hospitalization, and death, their ability to block infection and transmission remains limited, and protection wanes over time. Current heterologous booster strategies still rely largely on combinations of different parenteral vaccine platforms. Although these regimens can enhance systemic humoral and cellular immunity, they are insufficient to robustly induce upper respiratory mucosal immunity, thereby limiting their role in early infection control and interruption of transmission. Because respiratory mucosal vaccines target the natural entry site of SARS-CoV-2, they have the potential to induce secretory IgA, tissue-resident memory immune cells, and local immune memory, offering distinct advantages in compensating for the shortcomings of conventional heterologous boosting. Combining heterologous boosting with respiratory mucosal delivery may further enhance the control of viral infection, early replication, and subsequent transmission while preserving systemic immunity, thereby providing more comprehensive immune protection for high-risk populations. However, this strategy still faces challenges related to mucosal barriers, delivery efficiency, adjuvant safety, manufacturing scale-up, and incomplete clinical evaluation frameworks. Future high-quality studies focusing on high-risk populations are needed to clarify its value in preventing infection, protecting against severe disease, and providing long-term protection.

## 1 Introduction

**Figure 1:**
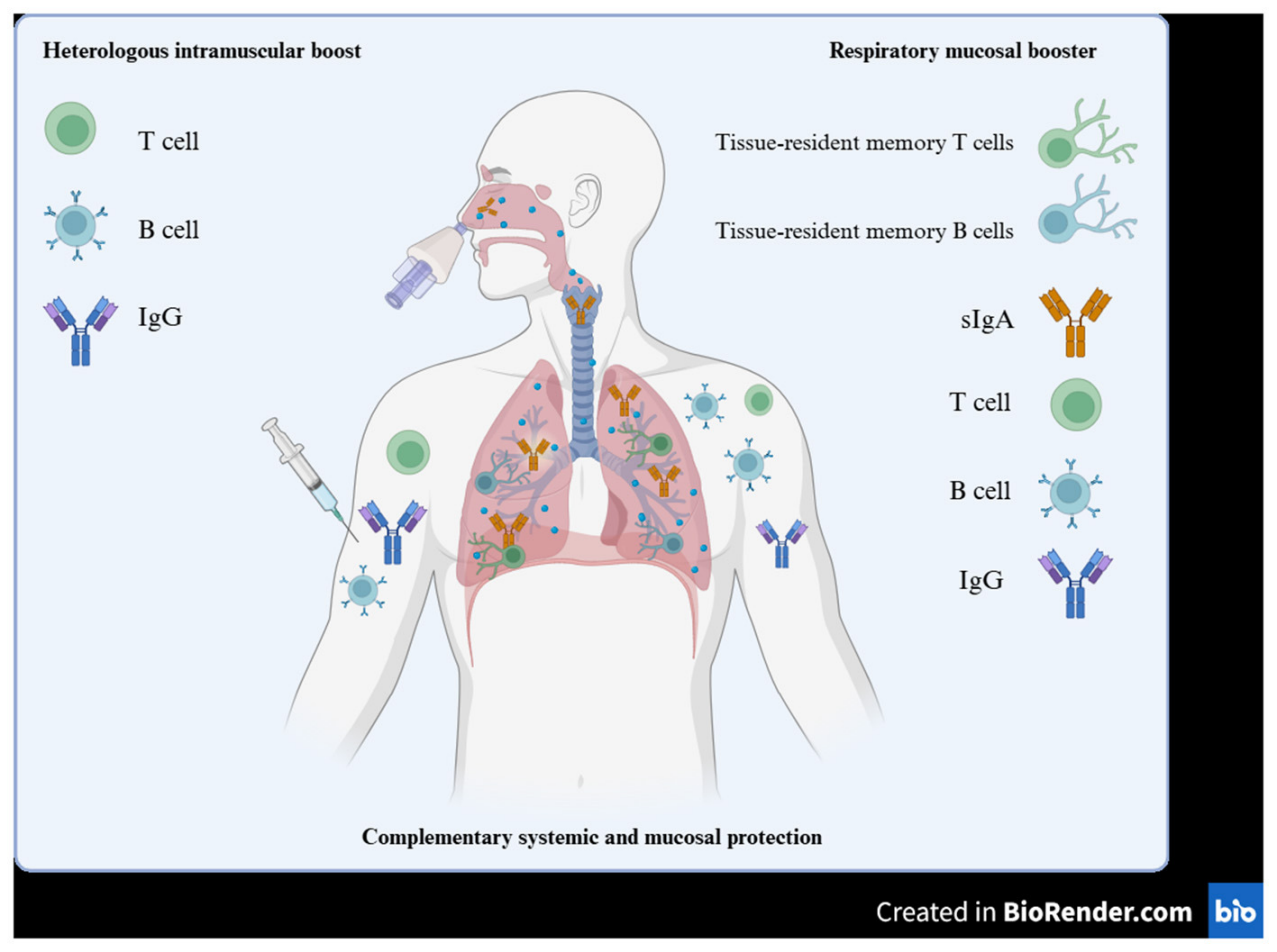
Schematic illustration of the immune responses induced by heterologous intramuscular boosting and respiratory mucosal delivery. Conventional heterologous booster regimens are typically administered by injection and primarily elicit systemic immune responses, characterized by circulating B cells, antiviral T cells, and IgG antibodies, but do not efficiently induce mucosal immunity in the upper respiratory tract. In contrast, respiratory mucosal vaccines, administered via the mucosal route, such as intranasal sprays, have been shown to induce both systemic and mucosal immune responses. In addition to B cells and antiviral T cells, mucosal vaccination can induce secretory IgA(sIgA) and tissue-resident memory T and B cells, which reside in mucosal tissues and provide local protection at the sites of viral entry, particularly in the upper respiratory tract. The combination of heterologous boosting and respiratory mucosal delivery may therefore provide complementary systemic and mucosal immune protection and offer improved protection for high-risk populations. Created with BioRender.com (http://biorender.com/; accessed on April 20, 2026), and reproduced with permission from BioRender.

Although the global peak of the COVID-19 pandemic has largely passed, SARS-CoV-2 continues to spread. The continued evolution and antigenic drift within the Omicron lineage have significantly enhanced the immune-evasive capacity of SARS-CoV-2, resulting in a significant reduction in the neutralizing activity of serum from vaccinated COVID-19 patients, and a rapid decline in the protective efficacy against Omicron and other variants (1, 2). During the Omicron wave, vaccine-induced protection waned over time (1). Existing heterologous vaccination strategies have limited capacity to effectively induce upper respiratory mucosal immunity, and both viral evolution and immune waning are independently associated with an increased risk of reinfection (3). Consequently, primary infections and reinfections continue to occur. High-risk populations, including older adults, individuals with comorbidities, and immunocompromised individuals, often exhibit weaker vaccine-induced immune responses or more rapid waning of immunity and are therefore at greater risk of severe disease (4–7). In this context, although booster immunization can further reduce the risk of hospitalization, its protective effect also declines over time (8). Consequently, high-risk populations continue to face a persistent threat. Current evidence suggests that, among older adults, heterologous booster regimens may be more effective than homologous regimens in protecting against SARS-CoV-2 infection. For example, following a two-dose inactivated vaccine primary series, a heterologous booster regimen combining inactivated and mRNA vaccines provides better protection against SARS-CoV-2 infection than a homologous regimen employing solely inactivated vaccines (9). However, even after heterologous boosting, antibody titers in older adults remain lower than those in younger individuals, and age and underlying diseases may further compromise vaccine immunogenicity (5). In addition, although a third dose can further enhance protection, this effect is not durable, and protection against severe outcomes may also wane over time (8). Older individuals and those with multiple comorbidities show poorer serological responses to COVID-19 vaccines (6, 10, 11), and chronic kidney disease, chronic lung disease, diabetes, and other conditions further increase the risks of breakthrough infection and subsequent hospitalization (12). Although breakthrough infection after vaccination may act as an infection-driven mucosal immune boost in otherwise healthy individuals (13), this uncontrolled exposure should not be considered a safe or intentional immunization strategy for these vulnerable groups or for immunocompromised individuals, who remain at risk of hospitalization and severe outcomes (4, 12, 14). Thus, even after vaccination, individuals with common comorbidities should remain vigilant against infection. For those with moderate to severe immunocompromise, additional vaccination can provide some protection, but the overall magnitude of protection remains limited (4). In highly immunosuppressed populations such as kidney transplant recipients, attenuated responses or persistent non-response after boosting remain common; although heterologous boosting is safe and well tolerated, it is not significantly superior to homologous mRNA boosting (15). These observations suggest that, in high-risk populations, the challenge is not simply whether booster vaccination is needed, but how to optimize booster strategies to improve the quality of immune responses, prolong protection, and further reduce the risks of severe outcomes and infection. Current heterologous booster strategies are still largely based on combinations of different injectable vaccine platforms (16). These regimens primarily induce systemic immunity, but have limited ability to elicit mucosal immunity and to reduce the risks of infection and transmission (13). By contrast, respiratory mucosal delivery and natural infection can both induce robust mucosal immunity and are more conducive to establishing a local immune barrier against viral infection and transmission (17). Therefore, heterologous boosting through respiratory mucosal delivery, which mimics natural infection, when combined with intramuscular heterologous vaccination strategies, may provide more comprehensive immune protection for high-risk populations and may represent a promising approach to booster optimization. In summary, this review discusses the strengths and limitations of intramuscular heterologous booster strategies and respiratory mucosal delivery, as well as the potential advantages of combining them and their relevance for high-risk populations, in order to provide a rationale for optimizing heterologous vaccination strategies (Figure 1).

## 2 Advantages and limitations of heterologous booster strategies

Compared with homologous boosting, heterologous booster strategies can integrate the immunological advantages of different vaccine platforms and elicit stronger and broader humoral and cellular immune responses, including higher levels of binding and neutralizing antibodies as well as more robust T-cell responses, thereby enhancing neutralization against Omicron and other emerging variants, while maintaining an acceptable safety profile (18–20). Among Ad26.COV2.S-primed individuals, heterologous mRNA boosting can enhance spike-specific T-cell responses (19). Likewise, after primary immunization with inactivated vaccines, heterologous booster regimens, particularly those incorporating mRNA vaccines, markedly enhance humoral immunity and improve neutralizing activity against Omicron and other SARS-CoV-2 variants, although neutralization against Omicron remains lower than that against the ancestral strain or Delta (20). During the Omicron wave, heterologous boosting further improved protection against symptomatic SARS-CoV-2 infection and severe outcomes, including hospitalization (21). Nevertheless, these strategies still have important limitations. Although current heterologous regimens can induce broad systemic immunity and increase serum neutralizing antibody responses, protection against symptomatic infection remains incomplete and wanes over time (1, 21). Moreover, with the emergence of immune-evasive variants such as Omicron, the protective effects of heterologous booster regimens may be further compromised (20). In addition, their efficacy is influenced by platform type and regimen design, and not all heterologous regimens are consistently superior to homologous regimens (16, 18). A major unresolved limitation is that most current vaccination strategies still rely mainly on injectable primary and booster vaccination, thereby predominantly enhancing systemic humoral immunity, particularly pathogen-specific IgG and neutralizing antibodies, rather than durable IgA-dominant respiratory mucosal immunity (18, 22). Consistent with this concern, although intramuscular mRNA vaccination can transiently induce salivary neutralizing activity, this mucosal immune response is mediated mainly by IgG rather than IgA and therefore differs from the more typical IgA-dominant mucosal immunity observed after infection (23). Therefore, strategies relying solely on injectable boosters may be insufficient to effectively limit early viral replication in the upper respiratory tract and may be associated with the continued risk of breakthrough infection and onward transmission (22–24). Together, these limitations highlight the need for vaccination strategies that strengthen frontline mucosal immune defense. Combining respiratory mucosal delivery with conventional heterologous boosting has the potential to strengthen both local and systemic immune responses, thereby improving protection against emerging variants and immune escape. Importantly, even if sterile immunity is difficult to achieve, such strategies may still confer substantial benefits by reducing the risks of infection, transmission, severe disease, and hospitalization.

## 3 Advantages and limitations of respiratory mucosal delivery

The key advantage of respiratory mucosal delivery lies in its ability to target the respiratory mucosa, the natural entry site of SARS-CoV-2. This approach has the potential to induce both local mucosal and systemic immune responses and to establish a frontline immune barrier characterized by secretory IgA (sIgA), tissue-resident memory T (TRM) cells, and memory B and T cells (25–27). By complementing systemic immunity, respiratory mucosal vaccination may enhance the breadth and durability of immune protection. In the context of continuous SARS-CoV-2 evolution, this strategy may strengthen local immune defense in both the upper and lower respiratory tract, improve cross-variant protection, and contribute to reducing the risks of infection, transmission, severe disease, and hospitalization (26–28). It should be noted that respiratory mucosal delivery is not a single route but encompasses intranasal delivery, aerosolized or nebulized inhalation, and intratracheal delivery (17, 29). These routes differ in antigen deposition sites, the magnitude of local immune responses, and the types of immunity induced. Intranasal delivery primarily targets the nasal cavity and nasopharynx and is therefore more suitable for enhancing upper respiratory tract mucosal immunity, particularly sIgA responses (27, 28). By contrast, aerosolized or nebulized inhalation facilitates antigen delivery to the lower respiratory tract, including the lungs, thereby promoting local IgA responses, memory B and T cells, and TRM cell induction (26, 30). Intratracheal delivery provides a more direct approach for establishing local immune barriers in the lower respiratory tract. Compared with intramuscular and intranasal routes, Ad26 intratracheal boosting more strongly enhanced pulmonary mucosal humoral and cellular immune responses and provided near-complete protection after high-dose SARS-CoV-2 BQ.1.1 challenge (29). Studies in non-human primates have further shown that intranasal immunization can markedly induce mucosal IgA and cellular immune responses and protect against severe pulmonary pathology and death. Although its protective efficacy against Omicron is reduced, it still confers partial protection against severe disease (27, 28). Similarly, the nebulized dNS1-RBD vaccine has been shown to activate innate immunity, trained immunity, and TRM cells, providing broad protection against multiple SARS-CoV-2 variants in mouse and hamster models (26). In addition, compared with intramuscular injection, respiratory mucosal vaccines may offer the practical advantages of needle-free, non-invasive, and convenient administration, which could improve acceptability, facilitate mass vaccination, and further reduce infection, transmission, and related disease burden.

Respiratory mucosal delivery also has clear limitations. High-quality human evidence demonstrating robust mucosal immunity and effective blocking of infection and transmission remains limited. In addition, respiratory mucosal vaccines are highly dependent on formulation and delivery systems, and their immunogenicity can be influenced by multiple factors, including mucosal barriers, antigen stability, delivery devices, dose, and local deposition patterns (22). Although some heterologous booster regimens can induce strong systemic immunity, their immunogenicity is clearly platform-dependent, and the magnitude of immune responses varies across vaccine regimens. Therefore, in the context of continued Omicron circulation, respiratory mucosal delivery should not be viewed as a replacement for conventional intramuscular vaccination. Rather, it should be considered a complementary approach to existing heterologous booster strategies. An integrated strategy combining respiratory mucosal delivery with systemic booster vaccination may provide more comprehensive protection, particularly for high-risk populations.

## 4 Potential of combining heterologous boosting with respiratory mucosal delivery in high-risk populations

The principal advantage of combining heterologous boosting with respiratory mucosal delivery lies in the simultaneous induction of systemic and mucosal immune responses, thereby enhancing the strength, breadth, and durability of protective immunity and establishing a more comprehensive barrier against infection. This combined approach may also better counter Omicron immune escape and increase neutralizing antibody responses against SARS-CoV-2 variants. By targeting early and breakthrough infection, reducing transmission risk, and lowering the risks of severe outcomes such as hospitalization, this strategy may provide broader and more effective protection. Previous studies have shown that heterologous boosting with aerosolized Ad5-nCoV is safe and highly immunogenic in large populations, enhancing both systemic and mucosal immunity; compared with inactivated vaccines, it may provide more durable protection against Omicron subvariants and may delay infection (31). In non-human primates, mucosal boosting with a bivalent adenoviral-vectored vaccine delivered by either the intranasal route or an aerosol device after prior intramuscular mRNA priming, induced more durable airway IgG/IgA responses and lung-specific B- and T-cell responses, effectively controlled XBB.1.16 replication in both the upper and lower respiratory tract, and suggesting advantages over conventional intramuscular boosting in preventing breakthrough infection and limiting transmission (24). In mouse models, intramuscular mRNA priming followed by intranasal heterologous boosting induced robust mucosal and systemic immunity, effectively controlled viral replication in the upper respiratory tract, and more markedly suppressed lung inflammation associated with severe disease (32). The “Prime and Spike” (P&S) strategy further supports this concept by using an unadjuvanted intranasal spike booster after systemic priming to redirect pre-existing immunity toward the respiratory mucosa. This strategy induced resident memory B cells, TRM, and mucosal IgA/IgG responses, while maintaining systemic neutralizing antibodies comparable to intramuscular mRNA boosting (25). In animal models, this strategy also enhanced viral control in both the upper and lower respiratory tract and may help limit transmission (25).

For high-risk populations, including older adults, individuals with chronic comorbidities, and those with impaired immune function, the significance of this combined strategy may be even greater. These populations generally exhibit weaker vaccine-induced immune responses, more rapid waning of protection, and a persistently higher risk of severe disease after breakthrough infection. Together with the limitations of existing vaccination strategies in durability of protection, interruption of infection and transmission, and prevention of severe clinical outcomes, these features further underscore the potential value of the combined approach in high-risk populations. Its advantages may broaden immune coverage against Omicron variants and enhance overall effectiveness, thereby better meeting the greater needs of high-risk populations for stronger, longer-lasting, and more comprehensive protection. In addition, the needle-free, non-invasive, and easily administered nature of respiratory mucosal vaccines may improve vaccine uptake, expand immune coverage, and reduce disease burden, giving this combined approach both theoretical advantages and practical potential as an optimized booster strategy for high-risk populations. A phase three trial of an intranasal mucosal vaccine showed overall good safety and tolerability in adults aged 60 years or older and in individuals with stable chronic diseases, although independent estimates of protection in these high-risk subgroups have not yet been reported (33). However, despite the promising theoretical rationale, findings from animal studies, and some early clinical data, human evidence on the use of COVID-19 mucosal vaccines in high-risk populations remains limited. Available published studies have focused primarily on the general adult population or healthy adults. This indicates that current evidence provides only preliminary support for the safety, immunogenicity, and a certain degree of protection of the strategy combining heterologous boosting with respiratory mucosal delivery in the general adult population. However, its independent protective efficacy, durability of protection, and risk–benefit profile have not yet been adequately validated in populations requiring a higher level of protection, including older adults, individuals with multiple chronic comorbidities, and those with moderate to severe immunocompromise. This further underscores the need for more in-depth investigation in this area.

## 5 Challenges and future perspectives

Although the global pandemic peak has passed, SARS-CoV-2 continues to circulate and evolve posing a persistent public health challenge. For older adults, individuals with underlying diseases, and immunocompromised populations, COVID-19 remains an important risk factor for severe disease and death. In addition, long COVID can affect the respiratory, cardiovascular, neurological, and other organ systems, with persistent adverse effects on daily functioning and quality of life, thereby further increasing individual and societal burden (34). Although existing heterologous booster strategies can enhance systemic antibody and cellular immune responses, they remain largely limited to combinations of different parenteral vaccine platforms and have limited ability to enhance upper respiratory mucosal IgA and local immune memory. In older adults and other high-risk populations, immunosenescence and disease-associated immune dysfunction further diminish the magnitude and durability of vaccine responses, making the long-term protective effects of current strategies less than ideal (4, 6, 14). Therefore, continued optimization of vaccination strategies remains essential for reducing infection burden and protecting high-risk populations. Existing clinical evidence suggests that combining heterologous boosting with respiratory mucosal delivery may preserve systemic immunity while further inducing mucosal immunity, enhancing local upper respiratory IgA, resident memory B/T cells, and rapid immune recall. This integrated strategy may more effectively suppress viral replication in the respiratory tract and, in some models, provide better transmission control than intramuscular boosting alone.

However, combining heterologous boosting with respiratory mucosal delivery still faces challenges. Respiratory mucosal delivery is hindered by multiple biological barriers, including low permeability and rapid clearance caused by the mucus barrier, epithelial tight junctions, and mucociliary clearance, as well as antigen degradation mediated by antibodies, proteases, and nucleases in mucus (22, 35). The tolerogenic mucosal environment and relatively inefficient antigen presentation may further limit effective immune activation (36). To overcome these limitations, current strategies mainly focus on optimizing adjuvants and delivery systems, including the use of mucoadhesive enhancers (such as chitosan and hydrogels), permeability enhancers (such as CT/LT and CTB nanoparticles), and immunostimulants (such as TLR agonists, poly(I:C), and STING agonists), with the aim of prolonging antigen retention, reducing degradation, and enhancing APC uptake and presentation (17, 37). However, the clinical translation of mucosal adjuvants remains constrained by safety concerns. Bacterial toxin-derived adjuvants, including cholera toxin (CT), heat-labile enterotoxin (LT), and their detoxified derivatives, are representative and potent mucosal adjuvants, but their use is limited by toxicity and neurological safety concerns (22, 38). In mice, intranasal administration of native CT damaged the olfactory system, whereas CTB and a non-toxic CT derivative did not show similar olfactory toxicity (38). LT-based formulations have raised similar safety concerns; for example, LTK63 has been associated with an increased risk of Bell's palsy, whereas the detoxified derivative LThαK showed favorable overall tolerability (22, 35). In addition, TLR/STING-based immunostimulants, including CpG/TLR9 agonists, can activate APCs and innate immune pathways; therefore, excessive immune activation, local inflammation, and systemic reactions should be carefully monitored during development (22, 39). Overall, safer mucosal adjuvant development requires rigorous safety assessment, long-term monitoring, population-specific risk evaluation, rational adjuvant design, and safe and effective delivery systems (22). Beyond these biological and immunological barriers, the translational feasibility of respiratory mucosal vaccines also depends on scalable manufacturing, cost, formulation stability, and compatibility existing vaccine delivery systems (40, 41). An ideal platform should be based on mature, scalable processes that enable rapid development while complying with current cGMP requirements, encompassing both the production of the drug substance (DS) and subsequent fill-and-finish operations (41). Adenoviral vectors, mRNA, and self-amplifying RNA (saRNA) platforms have demonstrated the potential for rapid large-scale production; however, their production costs differ markedly. Adenoviral vector platforms are constrained by high fixed costs and facility investments, whereas mRNA/saRNA platforms are primarily influenced by the costs of specialized raw materials and antigen doses, and the use of specialized delivery devices may further shift costs to the formulation and fill-and-finish stages (41) Formulation stability is particularly critical for respiratory vaccines. The composition of the formulation, nanoparticle characteristics, dosage form, and delivery device can all affect the immune response, while temperature fluctuations, shear stress, and thermal stress during storage and aerosolisation may compromise vaccine activity (40). Compared with liquid formulations requiring strict cold-chain conditions, freeze-dried, spray-dried, or dry-powder formulations can enhance thermal stability and facilitate distribution in resource-limited settings (42, 43). In addition, regulatory review of respiratory vaccines is generally more complex, particularly for drug-device combination products, which require additional evaluation of chemistry, manufacturing, and controls (CMC) and device performance. This increases development challenges and imposes higher demands on clinical trial design (17, 22). Furthermore, the immunological evaluation framework for respiratory mucosal vaccines remains insufficiently established. The clinical relevance of mucosal immune markers is still unclear, and established systemic correlates of protection, such as serum neutralizing antibodies, may not accurately reflect protective immunity at mucosal sites (22). Therefore, reliable mucosal correlates of protection should be established, and immunological sample collection and testing procedures should be standardized to support the clinical evaluation and further optimization of respiratory mucosal vaccines.

Beyond addressing these challenges, future research should not remain limited to evaluating safety and immunogenicity in the general adult population, but should instead focus on high-risk populations, including older adults, individuals with chronic comorbidities, and those who are immunocompromised. On the one hand, such studies should systematically assess the incremental benefit of the combined strategy of heterologous boosting and respiratory mucosal delivery in preventing infection, reducing transmission, lowering the risks of hospitalization and severe disease, and mitigating long-term sequelae. On the other hand, they should incorporate rigorous safety surveillance, long-term follow-up, and population-specific risk assessment. At the same time, antigen design should be continuously optimized against circulating variants, reproducible and quantifiable correlates of mucosal protection should be established, and direct comparisons with existing standard booster regimens are needed to clarify the clinical effectiveness and feasibility of this approach. Only after these key lines of evidence have been strengthened can the clinical value and public health significance of respiratory mucosal vaccines in high-risk populations be reliably defined, thereby informing future vaccine development and public immunization strategies.
